# Molecular detection and characterization of a novel Theileria genotype in Dama Gazelle *(Nanger dama)*

**DOI:** 10.1016/j.ijppaw.2023.04.002

**Published:** 2023-04-12

**Authors:** Ana Perez de Vargas, Shameem Habeeba, Mohd Farouk, Bakhita Al Hbabi, Amna Al Otaiba, Salama Al Muhairi, Zulaikha Al Hammadi, Asma Abdi Shah

**Affiliations:** a Life Sciences Department, Al Ain Zoo, United Arab Emirates; b Veterinary Laboratories Division, Abu Dhabi Agriculture and Food Safety Authority, United Arab Emirates

**Keywords:** Dama Gazelle, Theileria, Wildlife, Reservoir, Novel genotype

## Abstract

Wild animals play a critical role in maintenance and transmission of various tick-borne pathogens. It is essential to identify these wild host species that can serve as important reservoirs of tickborne diseases. In the present study we investigated Dama gazelle (*Nanger dama*) as a potential novel reservoir of Theileria spp. A total of 53 blood samples collected from Dama gazelle as part of the Al Ain Zoo preventive medicine program were screened for Theileria spp. by qPCR using a commercial assay, followed by additional studies using conventional PCR targeting an approximate 450-base pair (bp) fragment of the V4 hypervariable region of the 18S ribosomal RNA (rRNA) gene. Sequencing and phylogenetic analysis of a subset (20) of PCR amplicons revealed Theileria isolates from gazelles of Al Ain Zoo clustered closely to *Theileria* sp. Dama Gazelle (AY735115) from USA and were far away or did not cluster with the known *Theileria* spp. of ruminants namely *T. annulata, T. ovis, T. orientalis, T. luwenshuni, T.parva* and *T.sinensis*. Theileria genotypes detected in gazelles of present study were clearly distinct from the other common theileria species of ruminants. The present finding throws light on the critical role of reservoir host in maintenance and transmission of pathogen.

## Highlights

• First report of *Theileria* spp. in Dama gazelle (*Nanger dama*) in United Arab Emirates.
• Theileria genotypes detected were distinct from *Theileria* spp. seen in domestic herbivore species reported in MENA region.
• Wild host species can serve as important reservoirs of tickborne diseases.

## Graphical abstract

**Figure:**
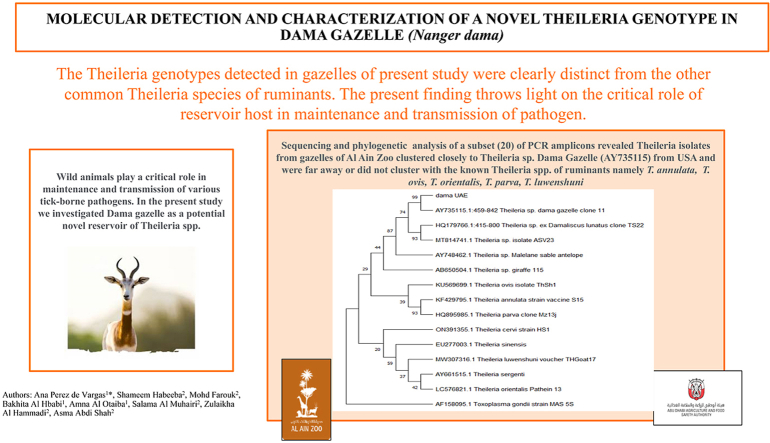

## 1 Introduction

Piroplasms belong to the most common group of mammalian blood parasites and their impact economically, as well as on veterinary and medical care, is significant (Jalovecka et al., 2018). Tick-borne diseases remain a major threat to the health of domestic and wild ruminants. *Theileria* spp. is one of the most economically important apicomplexan protozoans of the order Piroplasmida transmitted by hard ticks (Ixodidae) (Squarre et al., 2020). Tropical theileriosis is a common tick-borne disease of ruminants including cattle, sheep, and goats, and a major threat to the cattle industry causing serious illnesses with high morbidity and mortality rates, especially in naive animals. The two organisms with the greatest economic impact in cattle are *T. parva* and *T. annulata,* which cause East Coast fever/Corridor disease and tropical theileriosis, respectively whereas *T. lestoquardi, T.uilenbergi* and *T. luwenshuni* are the most virulent species in sheep and goats (Spickler and Anna., 2019). Wild animals serving as reservoir hosts for various tickborne infections has been well-established globally. They play a critical role in the maintenance of the endemic cycle of tickborne diseases, which increase the risk of disease transmission to humans and domestic animals due to their movements. Studies have detailed the diversity and distribution of Theileria and Babesia species in cattle (Bishop et al., 2004; Bock et al., 2004), and now with the advent of new molecular techniques, characterization of these organisms in wild herbivores are being taken up (Hooge et al., 2015).

Theileria has been reported from most of the countries in the Middle East and north African (MENA) region. Various tick species including *Hyalomma anatolicum* and *Hyalomma dromedarii* has been reported from United Arab Emirates (UAE) (Khan et al., 1997; Al-Deeb and Muzaffar, 2020). The DNA of *T. annulata* and *T. ovis* was detected using PCR in *H. anatolicum* ticks collected from cows and goats in Sharjah (the third largest emirate in UAE) (Perveen et al., 2021a). Multiple factors are involved in the increase in tick-borne diseases in the MENA region including expansion of tick geographic ranges due to wide-ranging live-stock farming, import of animals from other geographic regions, an abundance of wildlife populations that support ticks' lifecycles, climate change and improved diagnostics and surveillance (Perveen et al., 2021b). Different theileria species and novel theileria genotypes have been studied and reported in a wide range of wild ruminants including several species of deer (Inokuma et al., 2004; Garner et al., 2012; García-Sanmartín et al., 2007), Wildebeest (*Connochaetes taurinus*) (Wamuyu et al., 2015), tsessebe antelope (*Damaliscus lunatus lunatus*) (Brothers et al., 2011), wild sheep (*Ovis gmelinii anatolica*) (Orkun et al., 2016), waterbucks (*Kobus defassa*) (Pienaar et al., 2020), zebras (*Equus zebra*) (Githaka and Naftaly, 2014) and a few been studied particularly in wild gazelle species: Grant's gazelle (*Nanger granti*) (Hooge et al., 2015; Ghai et al., 2016) and Mongolian gazelle (*Procapra gutturosa*) (Youquan et al., 2014). The dama gazelle (*Nanger dama*) is one of the three most threatened antelope species. *Nanger dama* is listed as Critically Endangered under criteria C2a(i) by International Union for Conservation of Nature's (IUCN) Red List of threatened species with in-situ population numbers declining and very small and fragmented subpopulations due to the pressure of uncontrolled hunting, disturbance, and expanded livestock grazing (IUCN SSC Antelope Specialist Group, 2016). Dama gazelles are maintained in captive and semi-captive conditions in public and private facilities in America, Europe, and the Arabian Peninsula. Al Ain Zoo host an important captive population of this species, with 257 individuals recorded in March 2022. The species was historically split into three subspecies due to variations in coat coloration that are present in successive regions across its range: Mhorr (*N. d. mhorr*), Addra (*N. d. ruficollis*) and Dama (*N. d. dama*) (RZSS & IUCN Antelope Specialist Group, 2014). Two of these subspecies and its hybrids (part of a breeding experiment for meta-genomic population management planning) are present in Al Ain Zoo collection, Mhorr and Addra. The present study aimed a routine investigation of blood parasites to assess the health status of Dama Gazelles in the collection.

## 2 Materials and methods

The study was performed by analyzing 53 EDTA blood samples collected during scheduled veterinary preventive medicine procedures involving vaccination and health screening. All animals included in the study are descendants of Al Ain Zoo population (born in Al Ain Zoo) except for one male donated by other collection from UAE. The number of samples analyzed for each subspecies is detailed as follows: 21 Addra gazelle, 19 Mhorr gazelle and 13 hybrids. All animals were adults at the time of sampling except for one juvenile euthanized due to severe spinal injuries after intraspecific aggression. The animals are in different groups inside the zoo collection, and they have been relocated within the zoo over for breeding and husbandry purposes in the past. The DNA was extracted from all blood samples using QIAamp® DNA Mini kit as per manufacturer's instructions. The presence of Theileria piroplasm was investigated by Real Time PCR using the assay TheSpp MONODOSE dtec-qPCR kit (Genetic PCR Solutions™, Spain) following manufacturer instructions. For a detailed investigation on the strains of the piroplasmids circulating in wildlife, this was followed by amplification of 24 RT-PCR positive samples with the primers RLB-F2 (5′-GACACAGGGAGGTAGTGACAAG-3′) and RLB-R2 (5′-CTAAGAATTTCACCTCTGACAGT-3′) targeting an approximate 450-base pair (bp) fragment of the V4 hypervariable region of the 18S ribosomal RNA (rRNA) gene as described by Gubbels et al. (1999). One microlitre of the amplicon from this PCR step were then subjected to a second semi-nested PCR which used RLB-FINT (5′-GACAAGAAATAACAATACRGGGC-3′) as internal forward primer and RLB-R2(Schnittger et al., 2004). Amplicon from RLB-FINT/RLB-R2 were sequenced in SeqStudio Genetic Analyser (Thermo Fischer).

## 3 Results and discussion

**Fig. 1:**
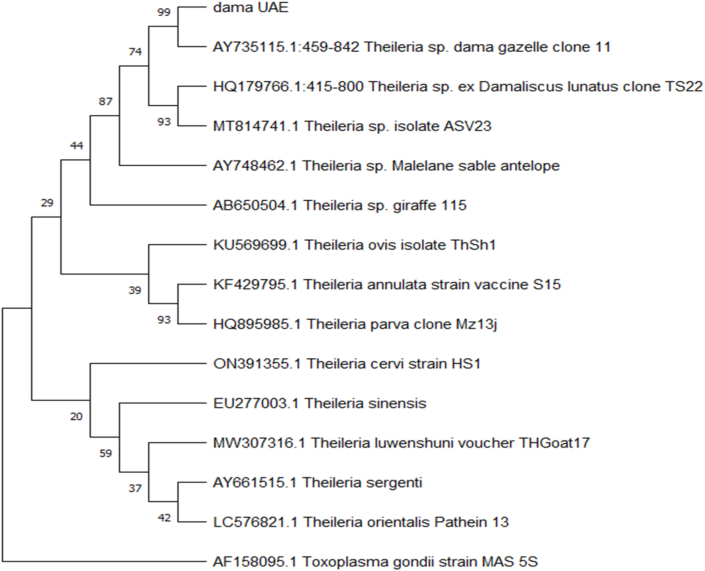
Phylogenetic analyses of sequence data for 393bp 18S rRNA gene of *Theileria* spp. in gazelles by Maximum Likelihood method with bootstrap of 1000 replications using MEGA software version10.

**Fig. 2:**
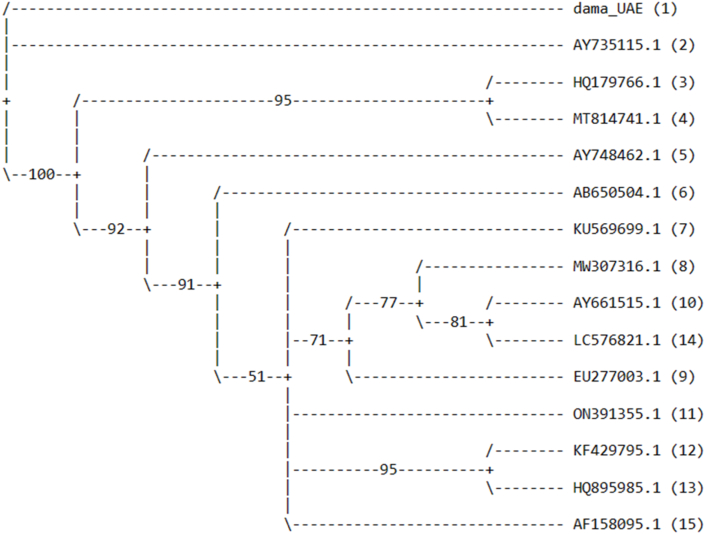
The clade credibility values of phylogram generated from bayesian analysis.

Phylogenetic relationships were assessed by using Maximum Likelihood (ML) method (Fig. 1) based on the kimira-2way model (Hornok et al., 2014) with the bootstrap resampling set at 1000 and the reliability analyses conducted in MEGA 10 (Kumar et al., 2016). 13 DNA sequences of 18s ribosomal RNA gene representative *Theileria* spp. were downloaded and *Toxoplasma gondii* used as an outgroup. Out of the 24 sequenced samples, a subset of 20 sequences were aligned using the clustalW. Since all sequences were of same identity, only one consensus used for the further analysis (dama UAE), aligned with the downloaded reference sequence and trimmed to 393 bp. The evolutionary tree was also generated using the Bayesian analysis (Fig. 2) with the parameter set to default and 1000 tree sampling from 10000 generated using Metropolis coupled MCMC. The reliability of the ML tree and credibility of Bayesian were compared to assess the agreement of the clade generated in the tree.

Our study documented the presence of a novel Theileria genotype in 20 Dama Gazelles from Al Ain Zoo (UAE) and revealed Theileria isolates from these gazelles clustered closely to *Theileria* spp. Dama Gazelle (AY735115) from USA and did not cluster with the known *Theileria* spp. of ruminants namely *T. annulata, T. ovis, T. orientalis, T. luwenshuni, T.parva* and *T.sinensis*. Thus, theileria genotypes detected in these gazelles were clearly distinct and far away from common *Theileria* spp. seen in domestic herbivore species reported in MENA region. These results were in accordance with earlier studies that reported variation in the 18S rRNA gene of various theileria species with extensive (1.5–1.7%) sequence heterogeneity even within the same clade grouping (Chaisi et al., 2013; Mans et al., 2015). 18S RNA is a highly conserved gene which enables genome analysis of differences between strains and species (Lack et al., 2012). Clade credibility and bootstrap reliability in our study showed that *T. sp*. Dama gazelle AA zoo and AY735115.1 share the same clade with 99% and 100% reliability and credibility. Theileria isolates detected in Grant's gazelles of Kenya represented two novel Theileria genotypes one clustered as a subgroup with previously identified *Theileria ovis* isolates from small ruminants from Europe, Asia and Africa whereas the second group clustered with previously identified *Theileria* spp. isolates from other African antelope (Hooge et al., 2015) and AY735115. Present phylogenetic study showed the study sequences clustered with isolates identified from several species of antelope (HQ179766, MT814741) and also revealed similarities (92%) with a *Theileria* sp. (AY748462) isolated from a sable antelope originating from Malelane (southern Kruger National Park area of South Africa). These similarities were confirmed by both neighbor-joining and bayesian phylogenetic approaches. No significant changes in the topology of the trees, or in the bootstrap values, were found in both phylogenetic analysis approaches.

It is suggested that gazelles occupy a central role in multi-species interaction networks which facilitate cross-species parasite transmission (Hooge et al., 2015). This feature of gazelle behavior may also increase the opportunity for parasite recombination events to occur in tick vectors resulting in minor levels of Theileria genome heterogeneity (Aktas et al., 2007; Katzer and Frank, 2011). In our study, none of the animals were showing any signs of disease at the time of collection and ticks were not found during physical inspection of the animals; body condition scores and temperature were normal, and none of them had any visible enlargement of the lymph nodes. Even though all gazelles had positive results, none of them had any clinical signs which poses a silent threat to other livestock by acting as a source of novel parasites. This lack of overt signs of pathogenicity in hemoparasite infection in wildlife has been reported earlier (Oosthuizen et al., 2008; Pfitzer et al., 2011; Brothers et al., 2011). Detailed studies of effect of *Theileria* spp. infections in Gazelles are required to assess the effect on general health and fitness of these animals along with the impact on other ungulates. There are earlier reports of spillover of *Theileria* spp. from one host species to others associated with clinical consequences in ungulates (Hofle et al., 2004; Nijhof et al., 2005).

## 4 Conclusions

To the best of our knowledge, this study is the first to report of Theileria spp. in Dama gazelle in United Arab Emirates. Further studies, with increased number of samples, vectors, and other wildlife ungulate species, need to be done to understand the role and possible interactions that gazelles and other wild ruminants might play in the maintenance of the endemic cycle of tick-borne diseases and the risk of disease trans-mission to domestic animals, particularly in this region where widespread farming often include gazelle species which might facilitate the spread of the disease.
